# Underrepresented patient views and perceptions of personalized medication treatment through pharmacogenomics

**DOI:** 10.1038/s41525-021-00253-1

**Published:** 2021-11-01

**Authors:** Loren Saulsberry, Keith Danahey, Brittany A. Borden, Elizabeth Lipschultz, Maimouna Traore, Mark J. Ratain, David O. Meltzer, Peter H. O’Donnell

**Affiliations:** 1 Department of Public Health Sciences, The University of Chicago, Chicago, IL USA; 2 Center for Personalized Therapeutics, The University of Chicago, Chicago, IL USA; 3 Center for Research Informatics, The University of Chicago, Chicago, IL USA; 4 Committee on Clinical Pharmacology and Pharmacogenomics, The University of Chicago, Chicago, IL USA; 5 Department of Medicine, The University of Chicago, Chicago, IL USA; 6 Section of Hospital Medicine, Department of Medicine, The University of Chicago, Chicago, IL USA

**Keywords:** Health care, Genetics research

## Abstract

Within an institutional pharmacogenomics implementation program, we surveyed 463 outpatients completing preemptive pharmacogenomic testing whose genetic results were available to providers for guiding medication treatment. We compared views and experiences from self-reported White and Black patients, including education level as a covariate across analyses. Black patients were less confident about whether their providers made personalized treatment decisions, and overwhelmingly wanted a greater role for their genetic information in clinical care. Both groups similarly reported that providers asked their opinions regarding medication changes, but White patients were more likely (59% vs. 49%, *P* = 0.005) to discuss the impact of personal/genetic makeup on medication response with providers, and Black patients reported initiating such discussions much less frequently (4% vs. 15%, *P* = 0.037). Opportunities exist for enhanced communication with underrepresented patients around personalized care. Tailored communication strategies and development of support tools employed in diverse healthcare settings may facilitate pharmacogenomically guided medication treatment that equitably benefits minority patient populations.

## Introduction

Pharmacogenomic testing is increasingly being incorporated into clinical care as a means to improve drug prescribing, reduce drug inefficacy, and avoid adverse drug events (e.g., toxicity)^1–5^. Pharmacogenomics uses an individual’s unique genetic makeup to assess their potential response to medication treatment, and such tailoring of healthcare delivery is one aspect of modern personalized care. Patient sense of personalized care is a dimension included in existing doctor–patient relationship measurement tools^6^. These and other patient-reported measures have been used to assess quality of care^7,8^, and they are increasingly becoming the focus of national health insurance programs like Medicare and Medicaid, which serve high-risk and vulnerable populations^9^. Pharmacogenomics has been shown to have a significant positive effect on patient perceptions of personalized care and other dimensions of the doctor–patient relationship^10^. Evaluating patients’ experiences with pharmacogenomics as a part of their medical care is critical to determining the clinical utility of genomic medicine.

Patient views while receiving pharmacogenomically guided care demonstrate an understanding that pharmacogenomics can inform prescribing and help distinguish problematic prescriptions^11^. Similar to other areas of care delivery, patients also expressed a desire for physicians to show personal attention by taking time to listen and discuss pharmacogenomic considerations with them^11^. Pharmacogenomic testing may change patient views about the role of genetics in their medical care, and these shifts in perceptions following genotyping might relate to aspects of the patient–provider relationship. One study found that patients felt pharmacogenomic testing offered insights to their physicians on dosing^12^. Other studies demonstrated that patients’ perceptions of receiving personalized care were higher after physicians used pharmacogenomics information to guide medication changes^10^ and that use of genomic information during prescribing increased patient–provider communication alongside patient recall of medication changes made during clinical visits^13^. Patients also reported decreases in concerns about medications and experiencing greater confidence about taking medications following pharmacogenomics testing^12^.

Despite these seemingly positive implications, public willingness to participate in genetic testing to inform medical care and views about pharmacogenomics use in clinical settings varies across demographic groups^1,14,15^. African Americans, who are at greater risk for experiencing health disparities^16,17^ have been largely underrepresented in pharmacogenomic studies^18^. Though less likely to be included and participate in pharmacogenomic implementation studies, African American, or Black, patients have focused more on the positive benefits of personalized medicine (e.g., fewer side effects and less trial and error prescribing) than White patients^19^. A study found no differences between Whites and Blacks in their perceived benefits of personalized medicine, but, similar to other research, it showed variation between racial groups in concerns about the use of personalized medicine, with Blacks expressing greater concerns than Whites about privacy and discrimination based on the use of genetic information^20–22^. Communication is often overlooked when new approaches to personalized care are adopted. Monitoring the impact of emerging technologies on health communication within clinical care may be specifically relevant for improving pharmacogenomics care delivery to minority patients as evidence has shown that minorities, patients of poor health status, patients with less than a high-school education, and older patients rate their visits with physicians as less participatory^17^.

In this study, we aimed to explore views and perceptions of care received among genotyped White and Black patients participating in a large institutional pharmacogenomic implementation program. We hypothesized that patients’ attitudes and perceptions about pharmacogenomics with respect to their medical care would significantly differ based on self-reported race. We particularly focused on self-reported race to define the populations in our study as self-identified race is used to direct pharmacogenomic clinical guidelines as well as clinical decision-making more generally in healthcare settings. Our study provides the perspectives of African American patients enrolled in a pharmacogenomic implementation program, whose views to date have not been deeply considered within pharmacogenomics implementation studies. Our primary objective was to identify potential gaps in patient perspectives that might guide how to best tailor pharmacogenomics implementation into clinical care for African Americans at greater risk for health disparities.

## Results

### Study participants

Patient characteristics are summarized in Table 1. Significant differences between total groups of White and Black respondents were apparent for gender and education, as more Black patients reported their gender as female and having an educational attainment of high school or less/some college. Black patients were also more likely to report their health as fair/poor. An education gradient was observed whereby higher educational attainment (e.g., college graduate/advanced degree) was associated with a greater likelihood of reporting excellent/very good health. Additional analyses alternatively stratifying by educational attainment showed significant differences remained in gender and self-reported health status when comparing total groups completing high school or less/some college and college graduate/advanced degree (Supplementary Table 1).

**Table 1:** Patient characteristics by self-reported race and educational attainment (*N* = 463).

	Race						
	White			Black			
Educational attainment	Total^1^	HS or less/ some college	College graduate/ advanced degree	Total^2^	HS or less/ some college	College graduate/ advanced degree	*P*-value^a^
Total survey respondents [*N* (%)]	332 (72)	86 (26)	246 (74)	131 (28)	74 (56)	56 (43)	
Gender [*N* (%)]							
Female	138 (42)	44 (51)	94 (38)	94 (72)	51 (69)	43 (77)	<0.0001***
Age [*N* (%)]							
Mean (range)	60 (19–90)	63 (19–90)	59 (20–87)	61 (19–95)	61 (19–89)	61 (26–89)	
18–25 years	8 (2)	2 (2)	6 (2)	1 (1)	1 (1)	–	
26–39 years	26 (8)	3 (3)	23 (9)	9 (7)	6 (8)	3 (5)	
40–50 years	33 (10)	5 (6)	28 (11)	21 (16)	10 (14)	11 (20)	0.306
51–64 years	122 (37)	33 (38)	89 (36)	43 (33)	22 (30)	21 (38)	
65+ years	143 (43)	43 (50)	100 (41)	57 (44)	35 (47)	21 (38)	
Total surveys returned evaluating clinical visits (after enrollment) [*N* (%)]	790 (75)	207 (26)	583 (74)	265 (25)	145 (55)	119 (45)	
Surveys returned per patient (after enrollment) [*N* (%)]							
Mean (range)	2 (1–16)	2 (1–16)	2 (1–14)	2 (1–7)	2 (1–7)	2 (1–6)	
1	149 (45)	40 (47)	109 (44)	66 (50)	41 (55)	24 (43)	
2	73 (22)	17 (20)	56 (23)	30 (23)	15 (20)	15 (27)	0.512
3–4	75 (23)	18 (21)	57 (23)	26 (20)	12 (16)	14 (25)	
5+	35 (11)	11 (13)	24 (10)	9 (7)	6 (8)	3 (5)	
Self-reported health [*N* (%)]^b^							
Excellent/very good	479 (61)	101 (50)	378 (65)	105 (40)	42 (30)	63 (53)	
Good	235 (30)	73 (36)	162 (28)	108 (41)	63 (44)	45 (38)	<0.0001***
Fair/poor	68 (9)	30 (15)	38 (7)	48 (18)	37 (26)	10 (8)	
*N*	782	204	578	261	142	118	

Survey responses of patients with “Unknown” educational level are not shown. Percent values may not sum to 100% due to rounding effects. ^a^Pearson chi-squared tests comparing ^1^total sample of self-reported White respondents and ^2^total sample of self-reported Black respondents. ^b^*N* values reflect responses from all surveys returned not unique patients. ^*^P ≤ 0.05, ^**^P ≤ 0.01, ^***^P ≤ 0.001.

### Patient views and experiences with their providers

White and Black patients reported exceedingly high levels of satisfaction with health care providers participating in the pharmacogenomics clinical study with almost all (99%) patients from both self-reported racial groups indicating being very satisfied/somewhat satisfied with their clinical visits (Table 2). More than 90% of both self-reported racial groups (White and Black) rated their providers as excellent/very good in three domains pertaining to patient–provider interactions: (1) being interested in the patient as a whole person, (2) explaining things clearly to the patient, and (3) making a plan of action with the patient (Table 2). Patient perceptions of the type of care approaches providers took during treatment were similar between White and Black respondents. Over ninety percent of survey respondents from both racial groups agreed strongly/agreed somewhat with statements describing provider care as based on up-to-date evidence and adherent to clinical guidelines (Table 2).

**Table 2:** Patient experience with providers participating in a pharmacogenomics implementation program by self-reported race and educational attainment.

	Race						
	White			Black			
	Total^1^	HS or less/some college	College graduate/ advanced degree	Total^2^	HS or less/some college	College graduate/ advanced degree	
Survey measure/question	*N* (%)	*N* (%)	*N* (%)	*N* (%)	*N* (%)	*N* (%)	*P*-value^a^
Overall, how satisfied were you with your healthcare provider visit today. Would you say you were very satisfied, somewhat satisfied, somewhat dissatisfied, or very dissatisfied?							
Very satisfied/somewhat satisfied	774 (99)	203 (99)	571 (99)	259 (99)	139 (99)	119 (100)	0.273
*N*	783	204	579	260	140	119	
Please rate today’s provider visit. How was the provider at…?							
Being interested in you as a whole person…(Asking/knowing relevant details about your life and your situation; not treating you as “just a number”)							
Excellent/very good	765 (97)	199 (97)	566 (98)	257 (97)	140 (97)	116 (97)	0.739
*N*	785	206	579	264	144	119	
Explaining things clearly…(Fully answering questions; explaining clearly; giving you adequate information; not being vague)							
Excellent/very good	766 (98)	202 (98)	564 (98)	260 (98)	142 (99)	117 (98)	0.407
*N*	784	206	578	264	144	119	
Making a plan of action with you…(Discussing the options; involving you in decisions as much as you want to be involved; not ignoring your views)							
Excellent/very good	737 (94)	197 (96)	540 (93)	247 (95)	131 (93)	115 (97)	0.071
*N*	785	206	579	261	141	119	
Please indicate your agreement or disagreement with each of the following statements:							
My healthcare provider cares greatly about me and my medical health							
Agree strongly/agree somewhat	773 (99)	200 (99)	573 (99)	259 (99)	141 (99)	117 (99)	
Disagree strongly/disagree somewhat	1 (<1)	0 (0)	1 (<1)	1 (<1)	1 (1)	0 (0)	0.695
Not sure	4 (1)	2 (1)	2 (<1)	1 (<1)	0 (0)	1 (1)	
*N*	778	202	576	261	142	118	
My healthcare provider incorporates “personalized medicine” into my treatment decisions							
Agree strongly/agree somewhat	720 (94)	181 (92)	539 (95)	231 (90)	125 (90)	105 (91)	
Disagree strongly/disagree somewhat	5 (1)	2 (1)	3 (1)	0 (0)	0 (0)	0 (0)	0.010**
Not sure	38 (5)	13 (7)	25 (4)	25 (10)	14 (10)	11 (9)	
*N*	763	196	567	256	139	116	
I want my healthcare provider to make medical decisions based upon the most up-to-date medical and scientific evidence available							
Agree strongly/agree somewhat	752 (97)	194 (97)	558 (97)	249 (96)	132 (95)	116 (97)	
Disagree strongly/disagree somewhat	12 (2)	3 (2)	9 (2)	4 (2)	2 (1)	2 (2)	0.305
Not sure	8 (1)	2 (1)	6 (1)	6 (2)	5 (4)	1 (1)	
*N*	772	199	573	259	139	119	
My healthcare provider follows medical guidelines or standards of practice when making medical decisions about me							
Agree strongly/agree somewhat	741 (97)	193 (97)	548 (96)	249 (97)	137 (97)	111 (96)	
Disagree strongly/disagree somewhat	1 (<1)	0 (0)	1 (<1)	0 (0)	0 (0)	0 (0)	0.813
Not sure	24 (3)	5 (3)	19 (3)	9 (3)	4 (3)	5 (4)	
*N*	766	198	568	258	141	116	

*N* values reflect the number of surveys returned from each self-reported racial group regarding clinical visits, not individual/unique patients. Survey responses of patients with “Unknown” educational level are not shown. Percent values may not sum to 100% due to rounding effects. ^a^Pearson chi-squared tests comparing ^1^total sample of self-reported White respondents and ^2^total sample of self-reported Black respondents. ^*^P ≤ 0.05, ^**^P ≤ 0.01, ^***^P ≤ 0.001.

Greater than 80% of all respondents from both racial groups and all educational levels thought healthcare providers had the ability to select medications that might work better for some patients because of knowledge about genetic/DNA factors. Despite this understanding of pharmacogenomics as a type of personalized medicine, White and Black patient perceptions varied on whether their provider incorporated “personalized medicine” into treatment decisions (Table 2). The majority of all respondents, whether White or Black, reported that they agreed strongly/agreed somewhat that providers incorporated “personalized medicine” into their treatment decisions, yet Blacks were twice as likely as Whites to report being unsure about receiving personalized care (10% and 5%, respectively, *P* = 0.01). No significant differences between patients’ responses about the use of “personalized medicine” remained when comparisons were performed between aggregate groups differentiated by educational attainment alone (high school or less/some college vs. college graduate/advanced degree) instead of race (Supplementary Table 2).

**Fig. 1:**
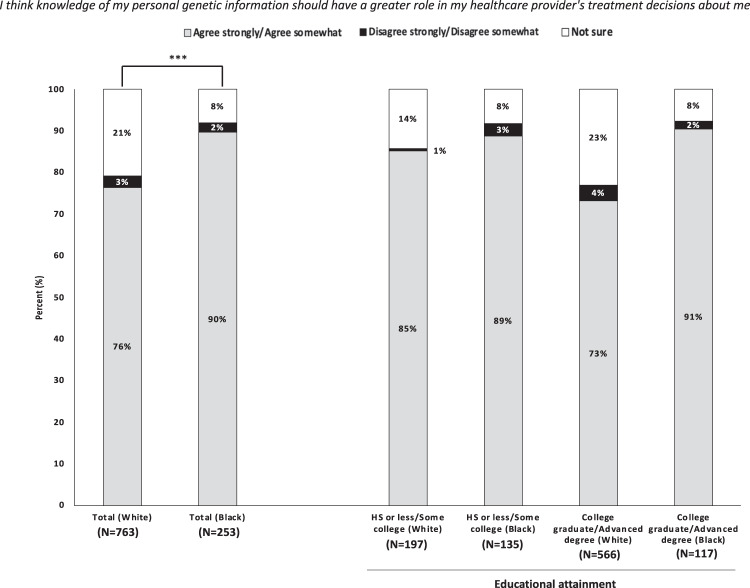
Patient views on the use of pharmacogenomics to guide health care delivery, by self-reported race and educational attainment. *N* values reflect the number of surveys returned from each self-reported racial group regarding clinical visits, not individual/unique patients. Survey responses of patients with “Unknown” educational level are not shown. Percent values may not sum to 100% due to rounding effects. Pearson chi-squared tests comparing total sample of self-reported White respondents and total sample of self-reported Black respondents. ^*^P ≤ 0.05, ^**^P ≤ 0.01, ^***^P ≤ 0.001.

Of all respondents, over 70% of respondents expressed receptivity for a greater role of personal genetic information in their clinical care (Fig. 1). Black patients (90%) were significantly more likely than White patients (76%) to report that they agreed strongly/agreed somewhat that their personal genetic information should play a greater role in their healthcare provider’s treatment decisions (*P* < 0.001). Black respondents in the aggregate and across education levels indicated similarly high levels of agreement that their personal genetic information should play a greater role in their healthcare provider’s treatment decisions (89% for high school or less/some college and 91% for college graduate/advanced degree). Such consistency in patient views was not observed across education levels for White respondents. White patients with higher educational attainment (college graduate/advanced degree) were less likely than those with high school or less/some college to agree that their personal genetic information should play a greater role in their healthcare provider’s treatment decisions for them (73% and 85%, respectively). This finding of the more highly educated patient population agreeing less frequently that their personal genetic information should play a greater role in their healthcare provider’s treatment decisions was similarly reflected in additional comparisons performed between total groups differing by educational attainment alone (high school or less/some college vs. college graduate/advanced degree) instead of race (Supplementary Fig. 1).

### Patient-reported participation in decision-making for medication changes during clinical visits

**Fig. 2:**
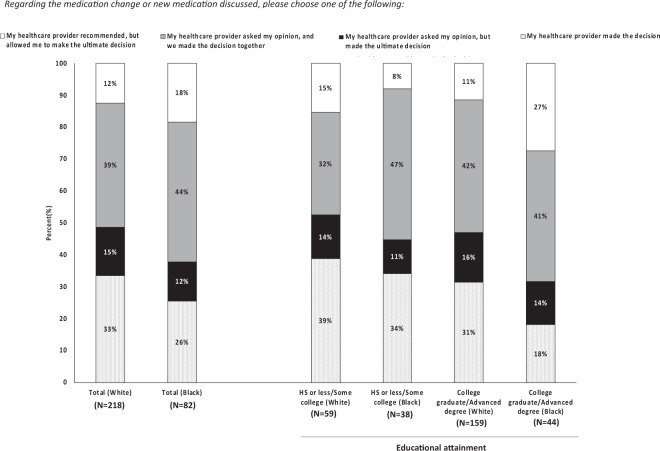
Patient-reported roles in decision-making for recalled medication changes made during a clinical visit, by self-reported race and educational attainment. *N* values reflect the number of surveys returned from each self-reported racial group regarding clinical visits, not individual/unique patients. Survey responses of patients with “Unknown” educational level are not shown. Percent values may not sum to 100% due to rounding effects. Pearson chi-squared tests comparing total sample of self-reported White respondents and total sample of self-reported Black respondents.

Before evaluating shared decision-making, we first importantly found that providers accessed GPS results at similar rates for evaluable health visits of Black and White study participants (69% vs. 72%, *P* = 0.317). Survey respondents expressed varying degrees of participation in the decision-making process about medication changes made during clinical visits (Fig. 2). More than half of White and Black respondents reported that their provider asked their opinion about a medication change or new medication. White patients with an education level of high school or less/some college were more likely to report that their provider made the decision about their medication change without patient consultation or input (39%). About 1 in 4 (27%) Black patients with higher educational attainment (college graduate/advanced degree) indicated that their provider made a recommendation but that they were allowed to make the ultimate decision about the medication change. This was almost double that of White patients from all educational backgrounds and nearly three times higher than the reported 8% of Black patients categorized as having lower educational attainment. The results did not differ when comparisons were performed between aggregate groups differentiated by educational attainment alone (high school or less/some college vs. college graduate/advanced degree) instead of race (Supplementary Fig. 2).

### Patient recollection of medication changes during clinical visits

Of all respondents, about 1 in 3 could remember their healthcare provider changing a medication during a clinic visit (Table 3). Recollection of medication changes was comparable between White (31%) and Black (33%) respondents (*P* = 0.27). Of the respondents that recalled a medication change, White and Black respondents significantly differed in reporting a discussion surrounding personalized medicine aspects of the medication decision with their healthcare provider. Black patients (49%) were significantly less likely than White patients (59%) to recall this discussion taking place, and Black patients were nearly three times more likely than White patients to be unsure whether they had this discussion with their providers (17% and 6%, respectively, *P* = 0.005). Although the majority of patients from both racial groups indicated that, if a personalized medication discussion occurred their provider initiated that conversation about individual factors in regard to medication changes, Black patients reported initiating these discussions much less frequently than White patients (4% vs. 15%, *P* = 0.037). Notably, not a single Black respondent with lower education attainment (high school or less/some college) reported initiating a discussion about personalized medicine with their provider. In subsequent analyses where survey responses were primarily stratified by educational attainment (high school or less/some college vs. college graduate/advanced degree) instead of race, racial differences persisted (Supplementary Table 3). That is, even among patients with lower educational attainment (high school or less/some college), White patients reported the highest rates of recalling provider discussions about personalized (genetic) factors, while 23% of Black patients compared to only 4% of White patients with the same lower educational attainment indicated being unsure about whether a personalized medicine discussion occurred with their provider.

**Table 3:** Patient recollection of medication changes during clinical visits with providers participating in a pharmacogenomics implementation program by self-reported race and educational attainment.

	Race						
	White			Black			
	Total^1^	HS or less/ some college	College graduate/ advanced degree	Total^2^	HS or less/ some college	College graduate/ advanced degree	
Survey measure/question	*N* (%)	*N* (%)	*N* (%)	*N* (%)	*N* (%)	*N* (%)	*P*-value^a^
Did your healthcare provider stop or change one of your medications today, or start a new medication?							
Yes	237 (31)	61 (31)	176 (31)	87 (33)	41 (28)	46 (40)	
No	532 (69)	136 (69)	396 (69)	170 (65)	101 (70)	68 (59)	0.273
Unsure	5 (1)	1 (1)	4 (1)	4 (2)	2 (1)	2 (2)	
*N*	774	198	576	261	144	116	
***If yes to Did your healthcare provider stop or change one of your medications today, or start a new medication*…**							
Did your healthcare provider discuss specific factors about you or your personal makeup which would suggest that you were more likely or less likely than other patients to benefit from the medication change or new medication?							
Yes	128 (59)	43 (75)	85 (53)	43 (49)	18 (46)	25 (52)	
No	77 (35)	12 (21)	65 (41)	29 (33)	12 (31)	17 (35)	0.005**
Unsure	12 (6)	2 (4)	10 (6)	15 (17)	9 (23)	6 (13)	
*N*	217	57	160	87	39	48	
*If yes, who initiated the discussion about individual factors regarding you and your response to the medication change or new medication?*							
I was the one who asked about individual factors	20 (15)	7 (16)	13 (15)	2 (4)	0 (0)	2 (7)	
My healthcare provider was the one who brought up individual factors	88 (67)	34 (76)	54 (63)	42 (86)	18 (86)	24 (86)	0.037*
Unsure	23 (18)	4 (9)	19 (22)	5 (10)	3 (14)	2 (7)	
*N*	131	45	86	49	21	28	

*N* values reflect the number of surveys returned from each self-reported racial group regarding clinical visits, not individual/unique patients. Survey responses of patients with “Unknown” educational level are not shown. Percent values may not sum to 100% due to rounding effects. ^a^Pearson chi-squared tests comparing ^1^total sample of self-reported White respondents and ^2^total sample of self-reported Black respondents. ^*^P ≤ 0.05, ^**^P ≤ 0.01, ^***^P ≤ 0.001.

## Discussion

In this study, we compared the views and experiences of self-reported White and Black patients receiving health care within a broad institutional pharmacogenomics implementation program. An overwhelming majority of Black patients wanted a greater role for their genetic information in their clinical care. While we found no disparities in satisfaction with provider care, there were gaps in the perceptions of White and Black patients in whether their providers were incorporating personalized medicine into treatment decisions. Compared to White patients, Blacks were less likely to express assurance that they were receiving personalized care from their providers despite having completed the same consent and enrollment process explaining the purpose of the pharmacogenomics implementation study. An association existed between self-reported race and patient uncertainty that was not observed when comparing responses primarily by education; proportional differences in reported uncertainty between Black and White patients within the same educational attainment group further supported this finding. White patients were more likely to report discussing the impact of personal makeup (genetics) on medication response with their provider, while Black patients reported initiating these discussions far less frequently. In fact, none of the self-reported lower educational attainment Black respondents in this study reported initiating such a discussion about personalized medicine with their provider.

Together, our results suggest an opportunity for enhanced patient–provider communication, especially for minority patients, around the role of genetic results during prescribing. This recapitulates findings from our own prior studies, which have demonstrated that the patient–provider relationship is critical to communication about pharmacogenomics^10,11,13^. The need for—and potential differential impact of—such discussions considering a patient’s race is a previously unappreciated aspect of this current work. Utilizing patient and physician “pairs” (where both parties agreed to undergo and receive, respectively, the genetic test results in this study) was an important feature of our pharmacogenomics model, with the expectation that it would lead to joint patient–provider decision-making, rather than the physician interpreting the information alone. Our results support this expected outcome as more than half of White and Black patients reported being asked their opinion about medication changes during clinical visits, with 40% reporting being asked their opinion and making the decision together with their provider.

Most genomic testing systems within US healthcare settings use provider-facing results portals (electronic health records; EHRs) to report results, thus (appropriately) requiring that communication about pharmacogenomic test results flows from provider to patient. Our implementation model was the same in this study (using the GPS, embedded within the EHR, to release results to providers). This amplifies the provider’s role as a pivotal gatekeeper of health information, especially pharmacogenomic risk information. Providers may be unprepared for the routine use of pharmacogenetic testing for clinical decision-making^23^ and even less equipped to adjust communication regarding pharmacogenomic information to underrepresented patient populations. As electronic patient portals evolve and patient access to that information technology expands, it is likely that patient demand will increase for using this communication medium to solicit consultation from providers about pharmacogenomics. Our results suggest that Black patients are currently not initiating these discussions with providers during health encounters. Further evaluation is needed to explain this lack of reported initiation and how those factors might bear upon pharmacogenomics implementation within underrepresented patient populations where these electronic tools are rapidly being incorporated into care delivery.

Given the significance of patient–provider communication in influencing patient behaviors and outcomes, tailored communication strategies may need to be developed and employed which address Black patients’ perceptions of receiving personalized treatments and help with the interpretation of pharmacogenomic information by providers for minorities and less educated patients. Prior studies evaluating patient engagement found that Blacks experience greater verbal passivity with physicians and lower levels of patient-centered communication compared to Whites and patients with higher educational attainment^17,24,25^. Our results similarly suggested that Black patients rarely initiated discussions regarding personalized medicine. Prior research also showed that racial minorities and patients with lower education lacked access to online patient portals or displayed limited use when they were available^26^. Few interventions exist that can simultaneously train patients to engage more fully in the healthcare process and give providers the skills needed to activate and promote patient engagement in the care dialogue^17^. These ideas illuminate the need for additional attention to developing racially and culturally sensitive tools to educate patients about genetic risks for suboptimal drug responses^27,28^.

This study is unique in that successful recruitment of Black patients within our institutional PGx implementation program permitted the consideration of both race and education in our analysis, expanding upon the current literature often treating racial groups as monolithic^29^. We recognize the differential role that education can serve as a more powerful determinant of health behaviors and outcomes for certain racial/ethnic groups than for others^30^. Education has also been associated with health literacy and understanding PGx test results^31,32^. Health literacy is defined by the Institute of Medicine (IOM) as the degree to which individuals can obtain, process, and understand the basic health information and services needed to make appropriate health decisions^33^. In our analysis, there was a larger representation of White patients with higher educational attainment (college graduate/advanced degree) compared to Black patients with a similar education level, replicating the education gradient frequently reported in the literature. Following stratification, we also observed a skewed distribution of gender across different education levels in both Black and White patient groups, illuminating the merit of further inquiry into the intersectionality of race and gender across educational backgrounds in influencing views and experiences with pharmacogenomics. The factors that make education influential in shaping health also intersect with race and gender, which influence social position. The intricacies of independent and/or interdependent contributions of these factors lack clarity and deserve to be actively pursued in future research.

In terms of thinking about whether personal genetic information should play a greater role in their provider’s treatment decisions, education appeared to (inversely) impact responses most among White patients. Given that educational attainment, which was associated with self-reported White race, can reflect the availability of greater resources and training to process health information, our finding that the more highly educated patient population agreed less frequently that their personal genetic information should play a greater role in their healthcare could represent more confident perceptions among the better educated that their genetic information is already being incorporated to maximally benefit them. In contrast, Black respondents across education levels indicated similarly high levels of agreement with this desire, suggesting that receptivity to personalized prescribing may be more broadly distributed across the Black patient population. Numerous mechanisms have been proposed through which education may shape views and perceptions of pharmacogenomic care. Prior research incorporating both race and education treat educational attainment as a proxy of socioeconomic status (SES) because it largely determines occupational status and income^30,34^. While pharmacogenomic testing was provided as a part of the clinical study free of charge to patients and providers, education might influence various aspects of access to healthcare, care delivery, and health outcomes that could be reflected in perceptions of pharmacogenomics.

This study had limitations. Our survey analysis includes responses from patients participating in one institutional pharmacogenomics implementation program at The University of Chicago Medical Center (UCMC) from 2012 to 2017, which may limit generalizability. While some changes to practice patterns may have occurred over time, dramatic changes since 2017 are unlikely as uptake of pharmacogenomics into regular clinical practice remains in the early stages at most academic centers and is not yet available in most community practices. Furthermore, the diffusion of pharmacogenomics into the healthcare delivered to minority patients has been limited. The diversity of our study cohort may strengthen the applicability of findings to future implementation in outpatient clinical settings and is specifically relevant to communication and patient experiences as pharmacogenomics is implemented into clinical care. The 1200 Patients Project incorporated patients receiving care from multiple types of outpatient providers across therapeutic areas, and UCMC’s location on the South Side of Chicago facilitated robust enrollment of Black patients to the clinical study, thus, to our knowledge, compiling one of the largest collections of first-hand accounts from Black patients of their views and experiences with pharmacogenomics implementation in the literature. Importantly, rather than utilizing survey measures to assess patient health literacy, we incorporated direct measures of educational status into the current study. The inclusion of direct measures assessing understanding of genetic test results is an area we are now exploring in other studies, before and after direct-to-patient delivery of pharmacogenomic test results^27^. Separately, though the absence of measures evaluating trust in providers/healthcare system among minority patients may be a limitation of some survey instruments, our survey instrument specifically measured trust, and our previous analyses of this cohort have reported that trust, defined as a patient’s belief that the provider had his or her best interest in mind when making clinical decisions, was generally high among our genotyped respondents^10^. Patients were asked to complete each survey immediately following their clinical visit, but in some instances survey responses were returned following a period of time after the visit, increasing the potential for recall bias. Finally, the total number of participating providers in this study was just under 20, so it is possible that some outcomes were driven by provider effects. However, our analyses did not account for physician clustering. Though providers included in the study were actively interested in pharmacogenomics, baseline provider knowledge about pharmacogenomics was modest, replicating the characteristics of more than 10,000 general US physicians^13^. Providers’ accessing GPS results was used as a proxy for disclosure. Since providers could elect to view and/or use pharmacogenomic results in their prescribing decisions, survey responses likely reflected authentic “patient–provider pair” interactions. The study design permitting inclusion of multiple surveys per patient over time for a more longitudinal view of patient experiences helped normalize any potential effect of prior knowledge.

As pharmacogenomics becomes more integrated into clinical practice and its use more widespread, its adoption is likely to be driven by both patients’ and providers’ understanding and interest in pharmacogenomic applications^15,35^. Future work should evaluate how point-of-care resources like the GPS, traditionally used for clinical decision support, might be leveraged to better equip both patients and providers with information and strategies that improve communication on how genetic results might influence medication treatment and response. Vigilance around the experiences of medically underserved populations will facilitate timely updating of approaches to deliver pharmacogenomically guided care that helps close communication gaps and incorporates patient views and preferences.

## Methods

### 1200 Patients Project

The 1200 Patients Project offered broad, preemptive pharmacogenomic testing to a diverse group of 1200 adults receiving outpatient subspecialty and primary care in order to assess the potential feasibility and utility of personalized medicine based on pharmacogenomics^36–38^. Patients and physicians were recruited into the study as pairs. Seventeen providers participated in this study, representing a diverse set of specialties across medicine (eight general internists, three oncologists, two cardiologists, one hepatologist, one nephrologist, one gastroenterologist, and one pulmonologist). Patients were then recruited to the 1200 Patients Project if they were receiving care from one of the participating physicians. Eligibility criteria were previously described^38^. The 1200 Patients Project recruited a racial/ethnic patient population that was approximately 60% White, 30% Black, and 10% Other^39^, reflecting the distribution of racial/ethnic populations in the greater Chicago city area^40^. While recruited patients were not given a formal education session on pharmacogenomics prior to survey completion, all patients received information describing the possible personalized care delivered in the study at the time of consent. These materials described personalized care as personal genetic information made available (with their permission) to their provider to enable specific medication treatment decisions for them, which aligns with our conceptualization of personalized care for the current study. Enrolled patients were genotyped across a broad panel of potentially actionable germline markers related to medication prescribing, with the pharmacogenomic information specific to each patient then shared with the patient’s physician through an online clinical decision support tool, the Genomic Prescribing System (GPS)^36–38^. The GPS provides patient-specific pharmacogenomic information at the point-of-care, and the medications included in the GPS with actionable pharmacogenomic information have been previously reported^36^. Our prior findings from the 1200 Patients Project indicated that 34% of all medications on patients’ active drug lists had associated pharmacogenomic results indicating genomically concordant medications (low-risk of toxicity and/or high chance for favorable response), genomically cautionary (increased risk of toxicity and/or suboptimal response), or genomically unfavorable (high-risk for toxicity and/or suboptimal drug response). A large majority (61%) of provider decisions to stop medications at clinic visits were influenced by pharmacogenomic recommendations. Relatedly, for new prescriptions, the decision to prescribe the chosen medication was affirmatively influenced by favorable pharmacogenomic information in half of all cases^39^.

### Study participants

The study setting consisted of clinical visits to outpatient healthcare providers. Following clinical visits, patients were issued surveys for completion and return to the research team. Indicators for issuing a survey to a patient in the 1200 Patients Project following a clinical visit have been previously described^10^. This study analyzed survey responses from self-reported White and Black patients enrolled within the 1200 Patients Project who completed genetic testing, were seen in follow-up, and experienced at least one clinical visit with a participating provider with access to the GPS clinical decision support tool during the study period from October 2012 to May 2017. Our final sample of survey responses included 463 participating patients: 332 patients who self-identified their race as White and 131 patients who self-identified their race as Black. A small number of patients (<12%) who identified as other races (i.e., not “White” or “Black”) were excluded from the present analysis.

### Survey instruments

Patients enrolled in the 1200 Patients Project were given anonymous surveys to complete after clinical visits with participating providers. At each clinical visit, providers could access patient pharmacogenomic results via the GPS, but the decision to view and/or use genetic information in their prescribing was at the providers’ discretion. Research staff independent of the providers gave surveys to patients after they saw their provider to complete before leaving clinic. If patients were unable to complete the surveys in the clinic, they were mailed the survey within a week of the patient’s visit. Over 95% of the surveys included in this analysis were completed immediately post visit, in the clinic. Less than 5% of all surveys received were by mail reporting.

The patient survey instrument was designed for evaluating five dimensions of the doctor–patient relationship, including trust, privacy, empathy, medical decision-making, and personalized care, as previously described in detail^10^. Development and pre-testing of the instrument was performed with the University of Chicago Survey Lab and members of the University of Chicago Center for Health and the Social Sciences, and with input from additional external reviewers. Pre-testing of drafts of the instrument was then performed institutionally by members of the research team, with iteration until the final version was achieved. The final survey instrument has been previously published and data/findings from its use have been previously reported for other cohort analyses^10,13^. For the current study, we specifically focused on the survey items surrounding patient perceptions of pharmacogenomic care and views regarding the use of genetic information to guide medication treatment.

Collectively, our final cohort of 463 White and Black patients completed 1055 surveys meeting this criteria (Table 1). Patient surveys were included in the analysis if at least half of the questions were completed (only 5 surveys were excluded from this analysis due to incomplete data). Questions that were left blank or answered inappropriately (e.g., multiple responses when only one was requested) were excluded from analysis of that item. While the survey domains remained the same throughout the study, we expected that patient perceptions of care could change over time because the prospective design of the 1200 patients study meant providers could variably choose to access pharmacogenomic results (or not) at each patient visit. Therefore, since each survey completed probed patient views and perceptions specific to that clinical visit, all surveys returned meeting inclusion criteria were evaluated, including those instances where the same patient returned multiple surveys over time because of having multiple (longitudinal) clinical visits. A prior evaluation showed an increase in providers’ referencing of pharmacogenomic results over time that influenced patient–provider communication^13^, so we predicted a similar impact could be reflected in patient perceptions of their numerous visits during the study. The median number of clinic visits was 2 across all patients, and those with more than 1 visit averaged a visit approximately every 8 months. Since provider visits triggered survey administration, these figures also indicate how far apart surveys were administered.

### Analysis plan

Our primary analysis compared patients in “White” and “Black” self-reported racial groups in the aggregate to learn how attitudes and perceptions might differ between the two groups. The prespecified analysis plan permitted the inclusion of multiple surveys collected over time for a single individual. This analytical approach involved treating each patient survey as an independent, additional observation evaluating a separate, distinct clinical visit. Since both Black and White patient groups had almost identical survey frequencies and response rates (returning on average 2 surveys/patient over the course of the study [Table 1]), we did not inversely weight the surveys by the number per subject. Survey responses to specific questions (measures) were compared using chi-square tests with the level of statistical significance set at *P* < 0.05 without adjustment for multiple comparisons.

While multiple theories have been developed to explain the crystallization of views/attitudes about health that relate to individual health behaviors, we undertook this analysis with education as a key covariate because it reflects the availability of resources (e.g., income and health insurance), the ability to process various types of information, and multiple socioeconomic indicators, including social and cultural factors^41^. Frameworks developed to foster health equity incorporate these factors in order to integrate context and promote the equitable diffusion of innovations^42^. This study included education as a covariate and performed secondary analyses of primary race-based comparisons by educational attainment to further our understanding of the respective roles of race and education. After performing the primary race-comparison analyses, we re-performed the analyses stratified by educational attainment within each self-reported racial group. For these analyses, educational level was categorized in a binary fashion: (1) “lower educational attainment” included patients reporting having completed high school or less, or some college; and (2) “higher educational attainment” included patients reporting being a college graduate or having an advanced degree. To further delineate the roles of race and education in our study, we also conducted sensitivity analyses of survey measures by also comparing results primarily by educational attainment, and, then, performing secondary analyses stratified by self-reported race within each educational attainment group (see online Supplement/Supplementary Information). Finally, as the GPS results were disclosed to patients at the discretion of the physician, we also assessed whether providers differentially accessed GPS results at health visits for Black and White patients within our final study sample.

### Ethics declaration

The 1200 Patients Project was an IRB-approved clinical study open at The University of Chicago (clinicaltrials.gov #NCT01280825), and all participants signed written informed consent.

### Reporting summary

Further information on research design is available in the Nature Research Reporting Summary linked to this article.

## Supplementary information

Supplementary information.

Reporting summary.

## Data Availability

All data generated or analyzed during this study are included in this published article and its supplementary information files.

## Code availability

Survey analyses were completed in Stata Statistical Software: Release 17 (College Station, TX: StataCorp LLC); the code and/or scripts underlying this article will be shared on reasonable request to the corresponding author.
