# Supplementary material for: Underrepresented patient views and perceptions of personalized medication treatment through pharmacogenomics
Source: NPJ Genom Med. 2021 Nov 1;6:90. doi: 10.1038/s41525-021-00253-1 (PMC8560901; doi:10.1038/s41525-021-00253-1)
Supplement: Supplementary file 1 — Supplementary information. [file 41525_2021_253_MOESM1_ESM.pdf]

Supplementary Table 1. Patient characteristics, by educational attainment and self-reported race (N = 462)

	Educational attainment						
	HS or less/Some college			College graduate/Advanced degree			
**Characteristics**	**Total[a]**	**White**	**Black**	**Total[b]**	**White**	**Black**	**p-value†**
Total survey respondents [N(%)]	160(35)	86(54)	74(46)	302(65)	246(81)	56(19)	
Gender [N(%)]							
Female	95(59)	44(51)	51(69)	137(45)	94(38)	43(77)	0.010**
Age [N(%)]							
Mean (range)	62(19-90)	63(19-90)	59(20-87)	59(20-89)	61(19-89)	61(26-89)	
18-25 years	3(2)	2(2)	1(1)	6(2)	6(2)	0(0)	
26-39 years	9(6)	3(3)	6(8)	26(9)	23(9)	3(5)	
40-50 years	15(9)	5(6)	10(14)	39(13)	28(11)	11(20)	0.689
51-64 years	55(34)	33(38)	22(30)	110(36)	89(36)	21(38)	
65+ years	78(48)	43(50)	35(47)	121(40)	100(41)	21(38)	
Total surveys returned evaluating clinical visits (after enrollment) [N(%)]	352(33)	207(59)	145(41)	702(67)	583(83)	119(17)	
Surveys returned per patient (after enrollment) [N(%)]							
Mean (range)	2(1-16)	2(1-16)	2(1-14)	2(1-7)	2(1-7)	2(1-6)	
1	81(51)	40(47)	41(55)	133(44)	109(44)	24(43)	
2	32(20)	17(20)	15(20)	71(24)	56(23)	15(27)	0.662
3-4	30(19)	18(21)	12(16)	71(24)	57(23)	14(25)	
5+	17(11)	11(13)	6(8)	27(9)	24(10)	3(5)	
Self-reported health [N(%)]‡							
Excellent/Very good	143(41)	101(50)	42(30)	441(63)	378(65)	63(53)	
Good	136(39)	73(36)	63(44)	207(30)	162(28)	45(38)	<0.0001***
Fair/Poor	67(19)	30(15)	37(26)	48(7)	38(7)	10(8)	
N=	346	204	142	696	578	118	

Percent values may not sum to 100% due to rounding effects. ‡ N-values reflect responses from all surveys returned not unique patients. † Pearson chi-squared tests comparing total sample of respondents with educational attainment of High school or less/Some college (a) and total sample of respondents with educational attainment of College graduate/Advanced degree (b). * *P*≤.05. ***P*≤.01. ****P*≤.001.

Supplementary Table 2. Patient experience with providers participating in a pharmacogenomics implementation program, by educational attainment and self-reported race

	Educational attainment						
	HS or less/Some college			College graduate/Advanced degree			
	Total[a]	White	Black	Total[a]	White	Black	
**Survey measure/question**	**N(%)**	**N(%)**	**N(%)**	**N(%)**	**N(%)**	**N(%)**	**p-value†**
Overall, how satisfied were you with your healthcare provider visit today. Would you say you were very satisfied, somewhat satisfied, somewhat dissatisfied, or very dissatisfied?							
Very satisfied/Somewhat satisfied	342(99)	203(99)	139(99)	690(99)	571(99)	119(100)	0.676
N=	344	204	140	698	579	119	
Please rate today's provider visit. How was the provider at…?							
Being interested in you as a whole person… (Asking/knowing relevant details about your life and your stituation; not treating you as "just a number")							
Excellent/Very good	339(97)	199(97)	140(97)	682(98)	566(98)	116(97)	0.928
N=	350	206	144	698	579	119	
Explaining things clearly …(Fully answering questions; explaining clearly; giving you adequate information; not being vague)							
Excellent/Very good	344(98)	202(98)	142(99)	681(98)	564(98)	117(98)	0.975
N=	350	206	144	697	578	119	
Making a plan of action with you …(Discussing the options; involving you in decisions as much as you want to be involved; not ignoring your views)							
Excellent/Very good	328(95)	197(96)	131(93)	655(94)	540(93)	115(97)	0.431
N=	347	206	141	698	579	119	
Please indicate your agreement or disagreement with each of the following statements:							
My healthcare provider cares greatly about me and my medical health							
Agree strongly/Agree somewhat	341(99)	200(99)	141(99)	690(99)	573(99)	117(99)	
Disagree strongly/Disagree somewhat	1(<1)	0(0)	1(<1)	1(<1)	1(<1)	0(0)	0.985
Not sure	2(<1)	2(1)	0(0)	3(<1)	2(<1)	1(1)	
N=	344	202	142	694	576	118	
My healthcare provider incorporates "personalized medicine" into my treatment decisions							
Agree strongly/Agree somewhat	306(91)	181(92)	125(90)	644(94)	539(95)	105(91)	
Disagree strongly/Disagree somewhat	2(1)	2(1)	0(0)	3(<1)	3(1)	0(0)	0.521
Not sure	27(8)	13(7)	14(10)	36(5)	25(4)	11(9)	
N=	335	196	139	683	567	116	
I want my healthcare provider to make medical decisions based upon the most up-to-date medical and scientific evidence available							
Agree strongly/Agree somewhat	326(96)	194(97)	132(95)	674(97)	558(97)	116(97)	
Disagree strongly/Disagree somewhat	5(1)	3(2)	2(1)	11(2)	9(2)	2(2)	0.746
Not sure	7(2)	2(1)	5(4)	7(1)	6(1)	1(1)	
N=	338	199	139	692	573	119	
My healthcare provider follows medical guidelines or standards of practice when making medical decisions about me							
Agree strongly/Agree somewhat	330(97)	193(97)	137(97)	659(96)	548(96)	111(96)	
Disagree strongly/Disagree somewhat	0(0)	0(0)	0(0)	1(<1)	1(<1)	0(0)	0.900
Not sure	9(3)	5(3)	4(3)	24(4)	19(3)	5(4)	
N=	339	198	141	684	568	116	

Percent values may not sum to 100% due to rounding effects. N-values reflect responses from all surveys returned not unique patients. † Pearson chi-squared tests comparing total sample of respondents with educational attainment of High school or less/Some college (a) and total sample of respondents with educational attainment of College graduate/Advanced degree (b).

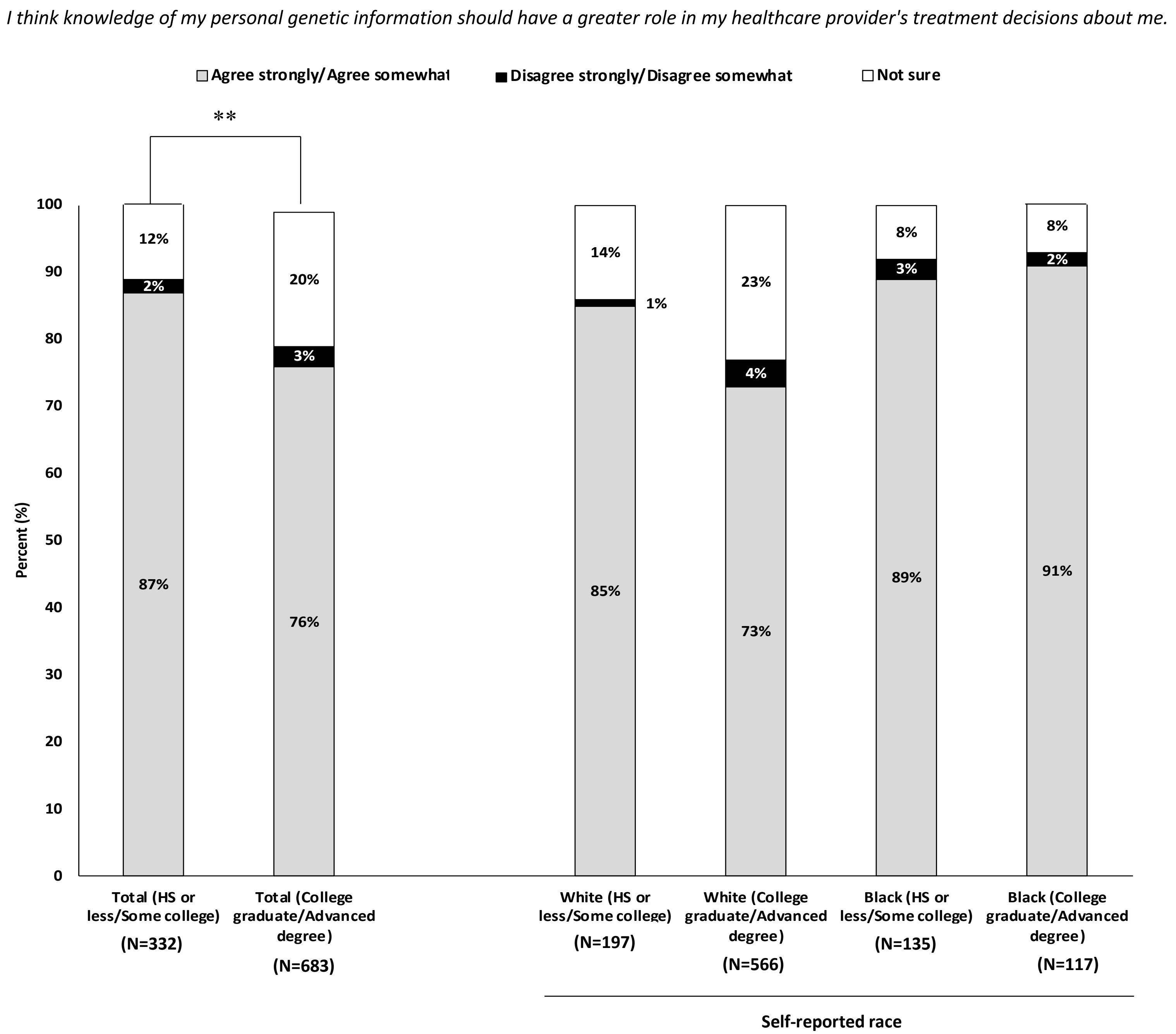

Supplementary Figure 1. Patient views on the use of pharmacogenomics to guide health care delivery, by educational attainment and self-reported race. N-values reflect the number of surveys returned from respondents of each educational attainment group regarding clinical visits, not individual/unique patients. Percent values may not sum to 100% due to rounding effects. Pearson chi-squared tests comparing total sample of respondents with educational attainment of High school or less/Some college (a) and total sample of respondents with educational attainment of College graduate/Advanced degree (b). **P*≤.05. ***P*≤.01.

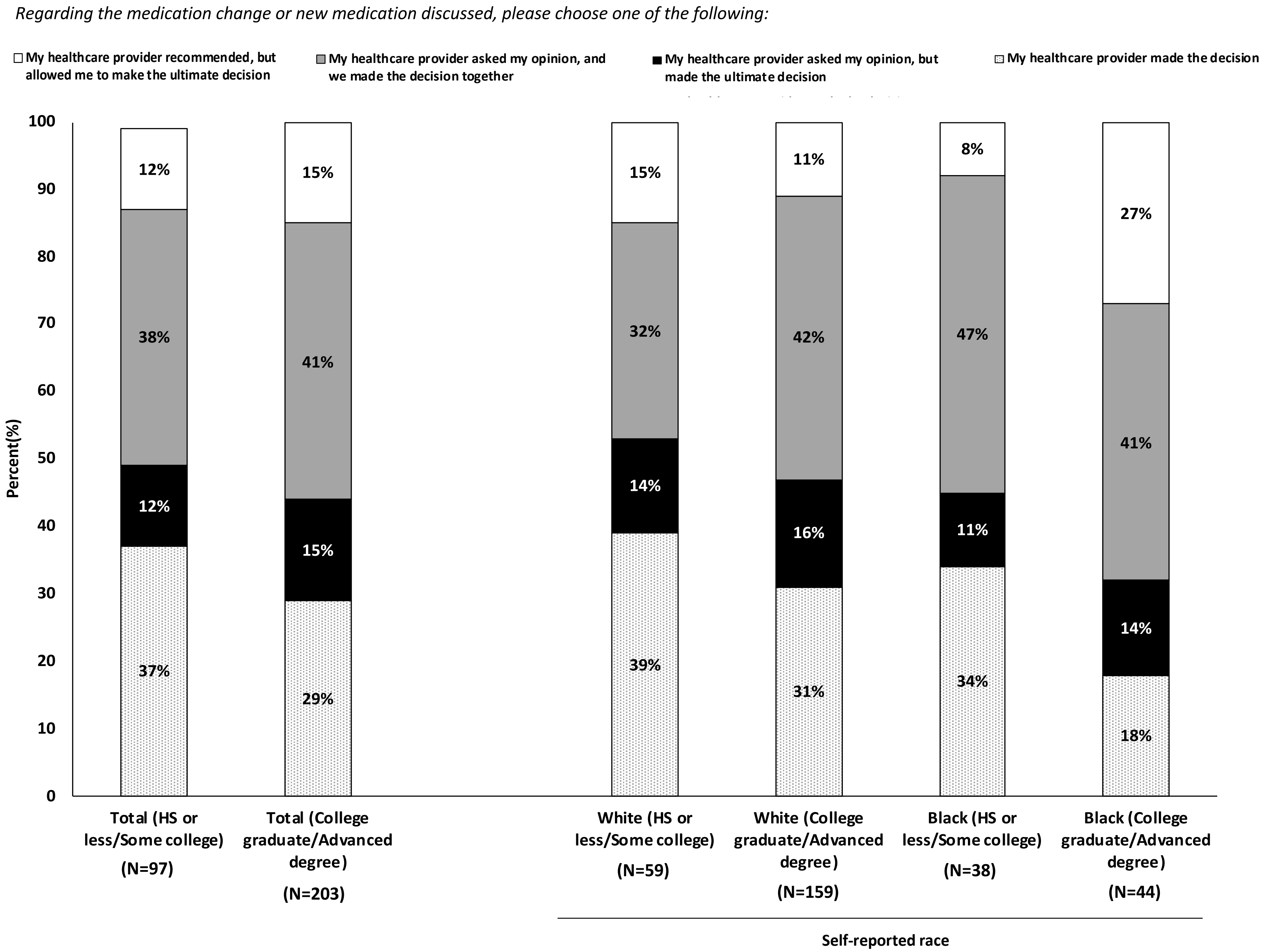

Supplementary Figure 2. Patient-reported roles in decision-making for recalled medication changes made during a clinical visit, by educational attainment and self-reported race. N-values reflect the number of surveys returned from respondents of each educational attainment group regarding clinical visits, not individual/unique patients. Percent values may not sum to 100% due to rounding effects. Pearson chi-squared tests comparing total sample of respondents with educational attainment of High school or less/Some college (a) and total sample of respondents with educational attainment of College graduate/Advanced degree (b).

Supplementary Table 3. Patient recollection of medication changes during clinical visits with providers participating in a pharmacogenomics implementation program, by educational attainment and self-reported race

	Educational attainment						
	HS or less/Some college			College graduate/Advanced degree			
	Total[a]	White	Black	Total[b]	White	Black	
Survey measure/question	N(%)	N(%)	N(%)	N(%)	N(%)	N(%)	p-value[†]
Did your healthcare provider stop or change one of your medications today, or start a new medication?							
Yes	102(30)	61(31)	41(28)	222(32)	176(31)	46(40)	
No	237(69)	136(69)	101(70)	464(67)	396(69)	68(59)	0.907
Unsure	3(1)	1(1)	2(1)	6(1)	4(1)	2(2)	
N=	342	198	144	692	576	116	
If yes to did your healthcare provider stop or change one of your medications today, or start a new medication…							
Did your healthcare provider discuss specific factors about you or your personal make-up which would suggest that you were more likely or less likely than other patients to benefit from the medication change or new medication?							
Yes	61(64)	43(75)	18(46)	110(53)	85(53)	25(52)	
No	24(25)	12(21)	12(31)	82(39)	65(41)	17(35)	0.043*
Unsure	11(11)	2(4)	9(23)	16(8)	10(6)	6(13)	
N=	96	57	39	208	160	48	
If yes, who initiated the discussion about individual factors regarding you and your response to the medication change or new medication?							
I was the one who asked about individual factors	7(11)	7(16)	0(0)	15(13)	13(15)	2(7)	
My healthcare provider was the one who brought up individual factors	52(79)	34(76)	18(86)	78(68)	54(63)	24(86)	0.289
Unsure	7(11)	4(9)	3(14)	21(18)	19(22)	2(7)	
N=	66	45	21	114	86	28	

N-values reflect the number of surveys returned respondents of each educational attainment group regarding clinical visits, not individual/unique patients. Percent values may not sum to 100% due to rounding effects.† Pearson chi-squared tests comparing total sample of respondents with educational attainment of High school or less/Some college (a) and total sample of respondents with educational attainment of College graduate/Advanced degree (b). * *P*≤.05.
