# Supplementary material for: Underrepresented patient views and perceptions of personalized medication treatment through pharmacogenomics
Source: NPJ Genom Med. 2021 Nov 1;6:90. doi: 10.1038/s41525-021-00253-1 (PMC8560901; doi:10.1038/s41525-021-00253-1)
Supplement: Supplementary file 2 — Reporting summary. [file 41525_2021_253_MOESM2_ESM.pdf]

nature portfolio

Corresponding author(s): Loren Saulsberry

Last updated by author(s): Sep 22, 2021

Reporting Summary

Nature Portfolio wishes to improve the reproducibility of the work that we publish. This form provides structure for consistency and transparency in reporting. For further information on Nature Portfolio policies, see our Editorial Policies and the Editorial Policy Checklist.

Statistics

For all statistical analyses, confirm that the following items are present in the figure legend, table legend, main text, or Methods section.

n/a	Confirmed	
☐	☒	The exact sample size (n) for each experimental group/condition, given as a discrete number and unit of measurement
☐	☒	A statement on whether measurements were taken from distinct samples or whether the same sample was measured repeatedly
☐	☒	The statistical test(s) used AND whether they are one- or two-sided<br>*Only common tests should be described solely by name; describe more complex techniques in the Methods section.*
☐	☒	A description of all covariates tested
☐	☒	A description of any assumptions or corrections, such as tests of normality and adjustment for multiple comparisons
☐	☒	A full description of the statistical parameters including central tendency (e.g. means) or other basic estimates (e.g. regression coefficient) AND variation (e.g. standard deviation) or associated estimates of uncertainty (e.g. confidence intervals)
☐	☒	For null hypothesis testing, the test statistic (e.g. F, t, r) with confidence intervals, effect sizes, degrees of freedom and P value noted<br>*Give P values as exact values whenever suitable.*
☒	☐	For Bayesian analysis, information on the choice of priors and Markov chain Monte Carlo settings
☒	☐	For hierarchical and complex designs, identification of the appropriate level for tests and full reporting of outcomes
☒	☐	Estimates of effect sizes (e.g. Cohen's d, Pearson's r), indicating how they were calculated

Our web collection on statistics for biologists contains articles on many of the points above.

Software and code

Policy information about availability of computer code

Data collection	Data and code availability statements included in manuscript.
Data analysis	Data and code availability statements included in manuscript.

For manuscripts utilizing custom algorithms or software that are central to the research but not yet described in published literature, software must be made available to editors and reviewers. We strongly encourage code deposition in a community repository (e.g. GitHub). See the Nature Portfolio guidelines for submitting code & software for further information.

Data

Policy information about availability of data

All manuscripts must include a data availability statement. This statement should provide the following information, where applicable:

- Accession codes, unique identifiers, or web links for publicly available datasets
- A description of any restrictions on data availability
- For clinical datasets or third party data, please ensure that the statement adheres to our policy

Data availability statement included in manuscript.

Field-specific reporting

Please select the one below that is the best fit for your research. If you are not sure, read the appropriate sections before making your selection.

☐ Life sciences ☒ Behavioural & social sciences ☐ Ecological, evolutionary & environmental sciences

For a reference copy of the document with all sections, see nature.com/documents/nr-reporting-summary-flat.pdf

Behavioural & social sciences study design

All studies must disclose on these points even when the disclosure is negative.

Study description	This study analyzed survey responses from self-reported White and Black patients enrolled within the 1200 Patients Project who completed genetic testing, were seen in follow-up, and experienced at least one clinical visit with a participating provider with access to the GPS clinical decision support tool. The study setting consisted of clinical visits to outpatient healthcare providers. Following clinical visits, patients were issued surveys for completion and return to the research team. This study was a quantitative survey analysis.
Research sample	Patients and physicians were recruited into the 1200 Patients Project study as pairs. Seventeen providers participated in this study, representing a diverse set of specialties across medicine. Patients were then recruited to the 1200 Patients Project if they were receiving care from one of the participating physicians. Our final sample of survey responses included 463 participating patients: 332 patients who self-identified their race as White and 131 patients who self-identified their race as Black. A small number of patients (<12%) who identified as other races (i.e., not 'White' or 'Black') were excluded from the present analysis.
Sampling strategy	This study was a retrospective survey analysis. The prespecified analysis plan permitted the inclusion of multiple surveys collected over time for a single individual. This analytical approach involved treating each patient survey as an independent, additional observation evaluating a separate, distinct clinical visit. Since both Black and White patient groups had almost identical survey frequencies and response rates (returning on average 2 surveys/patient over the course of the study [Table 1]), we did not inversely weight the surveys by the number per subject.
Data collection	Patients enrolled in the 1200 Patients Project were given anonymous surveys to complete after clinical visits with participating providers. At each clinical visit, providers could access patient pharmacogenomic results via the GPS, but the decision to view and/or use genetic information in their prescribing was at the providers' discretion. Research staff independent of the providers gave surveys to patients after they saw their provider to complete before leaving clinic. If patients were unable to complete the surveys in the clinic, they were mailed the survey within a week of the patient's visit. Over 95% of the surveys included in this analysis were completed immediately post-visit, in the clinic. Less than 5% of all surveys received were by mail reporting.
Timing	The study period extended from October 2012 to May 2017.
Data exclusions	Collectively, our final cohort of 463 White and Black patients completed 1,055 surveys meeting this criteria (Table 1). Patient surveys were included in the analysis if at least half of the questions were completed (only 5 surveys were excluded from this analysis due to incomplete data). Questions that were left blank or answered inappropriately (e.g. multiple responses when only one was requested) were excluded from analysis of that item. While the survey domains remained the same throughout the study, we expected that patient perceptions of care could change over time because the prospective design of the 1200 patients study meant providers could variably choose to access pharmacogenomic results (or not) at each patient visit. Therefore, since each survey completed probed patient views and perceptions specific to that clinical visit, all surveys returned meeting inclusion criteria were evaluated, including those instances where the same patient returned multiple surveys over time because of having multiple (longitudinal) clinical visits.
Non-participation	N/A; retrospective survey analysis
Randomization	N/A; retrospective survey analysis

Reporting for specific materials, systems and methods

We require information from authors about some types of materials, experimental systems and methods used in many studies. Here, indicate whether each material, system or method listed is relevant to your study. If you are not sure if a list item applies to your research, read the appropriate section before selecting a response.

Materials & experimental systems

n/a	Involved in the study	
☒	☐	Antibodies
☒	☐	Eukaryotic cell lines
☒	☐	Palaeontology and archaeology
☒	☐	Animals and other organisms
☐	☒	Human research participants
☒	☐	Clinical data
☒	☐	Dual use research of concern

Methods

n/a	Involved in the study	
☒	☐	ChIP-seq
☒	☐	Flow cytometry
☒	☐	MRI-based neuroimaging

Human research participants

Policy information about studies involving human research participants

Population characteristics	See above.
Recruitment	Patients and physicians were recruited into the study as pairs. Seventeen providers participated in this study, representing a diverse set of specialties across medicine. Patients were then recruited to the 1200 Patients Project if they were receiving care from one of the participating physicians. The study setting consisted of clinical visits to outpatient healthcare providers. Patients enrolled in the 1200 Patients Project were given anonymous surveys to complete after clinical visits with participating providers. The 1200 Patients Project recruited a racial/ethnic patient population that was approximately 60% White, 30% Black, and 10% Other, reflecting the distribution of racial/ethnic populations in the greater Chicago city area. Our survey analysis includes responses from patients participating in one institutional pharmacogenomics implementation program at The University of Chicago Medical Center (UCMC) from 2012-2017, which may limit generalizability, but the diversity of our study cohort may strengthen the applicability of findings to future implementation in outpatient clinical settings and is specifically relevant to communication and patient experiences as pharmacogenomics is implemented into clinical care. The 1200 Patients Project incorporated patients receiving care from multiple types of outpatient providers across therapeutic areas, and UCMC's location on the South Side of Chicago facilitated robust enrollment of Black patients to the clinical study, thus, to our knowledge, compiling one of the largest collections of first-hand accounts from Black patients of their views and experiences with pharmacogenomics implementation in the literature.
Ethics oversight	The 1200 Patients Project was an IRB-approved clinical study open at The University of Chicago (clinicaltrials.gov #NCT01280825), and all participants signed written informed consent.

Note that full information on the approval of the study protocol must also be provided in the manuscript.
